# Hospital Recorded Morbidity and Breast Cancer Incidence: A Nationwide Population-Based Case-Control Study

**DOI:** 10.1371/journal.pone.0047329

**Published:** 2012-10-19

**Authors:** Anne Gulbech Ording, Jens Peter Garne, Petra Mariann Witt Nyström, Deirdre Cronin-Fenton, Maja Tarp, Henrik Toft Sørensen, Timothy L. Lash

**Affiliations:** 1 Department of Clinical Epidemiology, Aarhus University Hospital, Aarhus, Denmark; 2 Breast Clinic, Aalborg Hospital, Aarhus University Hospital, Aarhus, Denmark; 3 Department of Oncology, Aalborg Hospital, Aarhus University Hospital, Aarhus, Denmark; 4 Department of Oncology, Uppsala University Hospital, Uppsala, Sweden; 5 Department of Epidemiology and Prevention, Wake Forest School of Medicine, Winston-Salem, North Carolina, United States of America; University of Hong Kong, China

## Abstract

**Introduction:**

Chronic diseases and their complications may increase breast cancer risk through known or still unknown mechanisms, or by shared causes. The association between morbidities and breast cancer risk has not been studied in depth.

**Methods:**

Data on all Danish women aged 45 to 85 years, diagnosed with breast cancer between 1994 and 2008 and data on preceding morbidities were retrieved from nationwide medical registries. Odds ratios (OR) and 95% confidence intervals (CI) were estimated using conditional logistic regression associating the Charlson comorbidity score (measured using both the original and an updated Charlson Comorbidity Index (CCI)) with incident breast cancer. Furthermore, we estimated associations between 202 morbidity categories and incident breast cancer, adjusting for multiple comparisons using empirical Bayes (EB) methods.

**Results:**

The study included 46,324 cases and 463,240 population controls. Increasing CCI score, up to a score of six, was associated with slightly increased breast cancer risk. Among the Charlson diseases, preceding moderate to severe renal disease (OR = 1.25, 95% CI: 1.06, 1.48), any tumor (OR = 1.17, 95% CI: 1.10, 1.25), moderate to severe liver disease (OR = 1.86, 95% CI: 1.32, 2.62), and metastatic solid tumors (OR = 1.49, 95% CI: 1.17, 1.89), were most strongly associated with subsequent breast cancer. Preceding myocardial infarction (OR = 0.89, 95% CI: 0.81, 0.99), connective tissue disease (OR = 0.87, 95% CI: 0.80, 0.94), and ulcer disease (OR = 0.91, 95% CI: 0.83, 0.99) were most strongly inversely associated with subsequent breast cancer. A history of breast disorders was associated with breast cancer after EB adjustment. Anemias were inversely associated with breast cancer, but the association was near null after EB adjustment.

**Conclusions:**

There was no substantial association between morbidity measured with the CCI and breast cancer risk.

## Introduction

Breast cancer is one of the most frequent cancers affecting women worldwide, with an estimated 1.38 million new cases diagnosed in 2008 [1]. Major breast cancer risk factors are sex and age [2]–[4], family history including BRCA1 and 2 mutations, oral contraceptives and postmenopausal hormone use [4], [5]. Other established risk factors are associated with endogenous sex hormones, such as reproductive history, lifestyle factors, physical inactivity, high postmenopausal body weight, and alcohol consumption [4], [6]. Only a fraction of all breast cancer cases can be explained by these risk factors, however [7].

Previous reports have identified diseases associated with breast cancer; yet no publication has exhaustively investigated a comprehensive set of diseases and their associations with breast cancer occurrence. Some suggested breast cancer mediators are estrogen-related diseases [8]–[12], some endocrine disorders [13], immune function [2], [3], [14], [15], inflammation [16], viral infections [17], and medication [18], [19]. Other diseases could be linked to breast cancer through various biologic or other underlying mechanisms, and the Charlson Comorbidity Index (CCI), which includes 19 disease categories weighted by their adjusted risk of one-year mortality [20], could be useful in measuring any combined effect of morbidities on breast cancer incidence.

We evaluated the associations between preceding morbidities, their complications, and subsequent breast cancer incidence using both the original [20] and an updated [21] CCI, and individual diseases included in the CCI [20]. As a hypothesis-screening analysis [22], we studied associations between an exhaustive set of preceding morbidities and subsequent breast cancer incidence. In a sub-analysis including only the breast cancer patients, we examined any association between morbidity and breast cancer stage at diagnosis.

## Methods

### Ethics Statement

The conduct of this study was approved by the Danish Data Protection Agency. In Denmark, no further permissions are needed to conduct registry-based studies such as our study. Informed consent from participants is therefore not needed.

### Source Population

We conducted this nested case-control study in a source population of all Danish women aged 45 to 85 years registered in the Danish Civil Registration System (CRS). Women with breast cancer diagnosed before 1 January 1994 were excluded. The CRS has collected information on date of birth, residence, and marital status for all Danish residents since 1968, when each was assigned a unique Civil Personal Registration (CPR) number encoding gender and date of birth. The CPR number is used in all Danish population and medical registries, and thus permits accurate individual-level linkage among registries [23].

The Danish Cancer Registry (DCR) has recorded national cancer incidence since 1943. It contains data on all cancers diagnosed through 2009 [24], [25]. The DCR used International Classification of Diseases (ICD)-7 codes until 2003 and have been converted to ICD-10 codes. Registration of breast cancer in the DCR is almost 100% complete [26].

All inpatient discharge diagnoses from non-psychiatric hospitals have been recorded in the Danish National Registry of Patients (NRP) since 1977. Outpatient data from all hospital departments and clinics were added in 1995. The DNRP records the CPR number and the date of each hospital visit, together with primary and secondary discharge diagnoses [27]. Diagnoses were coded according to ICD-8 from 1977–1993 and ICD-10 thereafter.

### Identification of Cases and Controls

We defined cases as all female patients aged 45 to 85 years who were diagnosed with incident breast cancer (ICD-10: C50) between 1 January 1994 and 31 December 2008 and registered in the DCR. Risk-set sampling without replacement was used to select 10 female controls without prevalent breast cancer from the source population, matched to each case by year of birth and calendar year. We defined the index date as the date of breast cancer diagnosis for cases and the date of the index case’s breast cancer diagnosis for controls.

### Data Collection

Date on breast cancer occurrence and stage at diagnosis were collected from the DCR. Data on all primary hospital diagnoses including the diseases in the CCI [20] up to 10 years before the index date were retrieved from the DNRP for each case and control. The CCI has been shown to be a valid prognostic marker of mortality in breast cancer patients [20]. It is based on selected disease categories that are weighted according to the adjusted one-year mortality risk [20]. It has recently been updated to reflect changes in survival due to medical advances and to administrative databases as a source of data [21]. Age was ascertained from the CRS. Because of the potential latency period preceding breast cancer diagnosis, we excluded all conditions registered for cases and controls in the three years preceding the index date.

### Analytic Variables

Age was categorized into five groups (45–50, 51–60, 61–70, 71–80, and 81–85 years). Morbidity was measured with the original [20] and an updated [21] CCI (scores of 0, 1, 2, 3, 4, 5, 6, 7, and ≥8) (Table S1). In the models associating CCI scores with breast cancer stage, CCI was categorized as 0, 1, 2, 3, and ≥4. Each individual disease in the CCI also was analyzed separately (presence/absence). Stage was categorized as local, regional, distant, and missing.

Based on the ICD-8 and ICD-10 World Health Organization morbidity tables [28], [29], we grouped all ICD-codes into 202 morbidity categories (Table S2), similar to categories previously used by our group [30]. We excluded from the analyses diagnoses reflecting external causes of morbidity (such as accidents) recorded during routine hospital outpatient visits and diagnoses only affecting men.

### Statistical Methods

We calculated distributions and frequencies of cases and controls by age at inclusion, index year, CCI score, and each of the 19 diseases included in the CCI. Contingency tables were constructed for each of the 202 morbidity categories. Conditional logistic regression models were used to calculate odds ratios (ORs) and 95% confidence intervals (CIs) associating breast cancer incidence with original and updated CCI scores, individual diseases included in the CCI, and each morbidity category within the risk-set matched strata. For the breast cancer patients, we used logistic regression models to calculate the OR for distant stage vs. local/regional stage breast cancer at diagnosis. CCI score in five categories (0, 1, 2, 3, and ≥4) and age as a continuous variable were included in the models as independent variables. Breast cancer patients with missing stage were excluded from this analysis.

In the hypotheses-screening analysis, the associations between 202 morbidity categories and breast cancer incidence were estimated. Given the study sample, these associations were not independent, leading to a statistical problem with type I errors, since the risk of obtaining 95% confidence intervals that do not contain the true population parameter by chance increases with the number of comparisons. The hypothesis-screening part of the study was conducted to identify both weak and strong associations, and had no a priori expectations of which comparison may be true. Confidence intervals centered far from the null may reflect unstable estimates of the true association, particularly when the interval is wide. The empirical-Bayes (EB) method shrinks the parameters toward the null association, taking into account the standard deviation of the original estimates. Estimates far from the null and imprecisely measured shrink the most, thereby de-emphasizing the associations most likely to be false-positives. Therefore, an EB method was applied to bring the size of the estimates and variances towards the overall mean and reduce the potential for spurious associations. To further stabilize the EB adjusted estimates, we excluded morbidity categories with fewer than five exposed cases. The assumptions behind the EB estimations, such as normality of the estimates, were satisfied [31].

All analyses were performed with SAS version 9.2 and Stata IC version 11.1.

## Results

The study included 46,324 breast cancer cases and 463,240 population controls. Table 1 presents the distributions of cases and controls categorized by age group and index year. For both the original and updated CCI, increasing scores up to a score of six were associated with slightly increased risk of breast cancer. Among the individual diseases included in the CCI and diagnosed three to ten years before the index date, moderate to severe renal disease (OR = 1.25, 95% CI: 1.06, 1.48), any tumor (OR = 1.17, 95% CI: 1.10, 1.25), moderate to severe liver disease (OR = 1.86, 95% CI: 1.32, 2.62), and metastatic solid tumors (OR = 1.49, 95% CI: 1.17, 1.89), were most strongly associated with subsequent breast cancer. Myocardial infarction (OR = 0.89, 95% CI: 0.81, 0.99), connective tissue disease (OR = 0.87, 95% CI: 0.80, 0.94), and ulcer disease (OR = 0.91, 95% CI: 0.83, 0.99) were most strongly inversely associated with subsequent breast cancer. Results based on the original and updated CCI are shown in Table 2, and results for the individual 19 diseases included in the CCI are shown in Table 3. The proportion of distant stage breast cancer increased with increasing CCI score and with the presence of some individual Charlson diseases. However, with logistic regression models adjusted for age, there was no association between comorbidity and breast cancer stage (Table 4).

**Table 1:** Frequencies and proportions of breast cancer cases and controls by age group and index years of diagnosis.

	Casesn = 46,324	(%^a^)	Controlsn = 463,240	(%^a^)
**Age group**				
45–50	4,815	(10)	48,494	(10)
51–60	13,273	(29)	132,469	(29)
61–70	13,924	(30)	139,025	(30)
71–80	10,020	(22)	100,269	(22)
81–85	4,292	(9.3)	42,983	(9.3)
**Index year**				
1994	2,659	(5.7)	26,590	(5.7)
1995	2,658	(5.7)	26,580	(5.7)
1996	2,787	(6.0)	27,870	(6.0)
1997	2,799	(6.0)	27,990	(6.0)
1998	2,880	(6.2)	28,800	(6.2)
1999	2,992	(6.5)	29,920	(6.5)
2000	3,036	(6.6)	30,360	(6.6)
2001	3,108	(6.7)	31,080	(6.7)
2002	3,300	(7.1)	33,000	(7.1)
2003	3,215	(6.9)	32,150	(6.9)
2004	3,174	(6.9)	31,740	(6.9)
2005	3,172	(6.9)	31,720	(6.9)
2006	3,322	(7.2)	33,220	(7.2)
2007	3,350	(7.2)	33,500	(7.2)
2008	3,872	(8.4)	38,720	(8.4)

a Because of rounding, percentages may not add to 100%.

b Controls were matched to cases on this variable.

**Table 2:** Original and updated Charlson Comorbidity Index (CCI) scores associated with breast cancer incidence among cases and controls.

Cases, n (%^a^)		Controls, n (%^a^)	OR (95% CI)
**Original CCI score**			
0	40,276 (87)	403,983 (87)	Ref
1	3,574 (7.9)	36,999 (8.0)	0.97 (0.94, 1.01)
2	1,781 (3.8)	16,650 (3.6)	1.08 (1.02, 1.13)
3	447 (1.0)	3,628 (0.8)	1.24 (1.12, 1.37)
4	129 (0.3)	1,076 (0.2)	1.21 (1.00, 1.45)
5	33 (0.1)	260 (0.1)	1.28 (0.89, 1.83)
6	65 (0.1)	452 (0.1)	1.44 (1.11, 1.87)
7	11 (0.02)	117 (0.03)	0.95 (0.51, 1.78)
≥8	8 (0.02)	75 (0.02)	1.01 (0.52, 2.22)
**Updated CCI score**			
0	42,423 (92)	426,147 (92)	Ref
1	1,834 (4.0)	19,071 (4.1)	0.97 (0.92, 1.02)
2	1,625 (3.5)	14,450 (3.1)	1.13 (1.07, 1.19)
3	243 (0.5)	2,147 (0.5)	1.14 (1.00, 1.30)
4	105 (0.2)	736 (0.2)	1.44 (1.17, 1.76)
5	17 (0.04)	131 (0.03)	1.31 (0.79, 2.17)
6	64 (0.1)	452 (0.1)	1.42 (1.10, 1.85)
7	5 (0.01)	49 (0.01)	1.03 (0.41, 2.58)
≥8	8 (0.02)	57 (0.01)	1.41 (0.67, 2.96)

Abbreviations. OR: Odds ratio.

a Because of rounding percentages may not add to 100.

**Table 3:** Individual diseases included in the Charlson Comorbidity Index (CCI) three to ten years preceding the index date and their association with breast cancer incidence.

Charlson disease	Exposedcases, n	OR (95% CI)
Myocardial infarction	432	0.89 (0.81, 0.99)
Congestive heart failure	485	1.19 (1.09, 1.31)
Peripheral vascular disease	524	1.01 (0.92, 1.10)
Cerebrovascular disease	1,098	1.04 (0.98, 1.10)
Dementia	91	0.88 (0.71, 1.09)
Chronic pulmonary disease	1,260	1.04 (0.98, 1.11)
Connective tissue disease	641	0.87 (0.80, 0.94)
Ulcer disease	525	0.91 (0.83, 0.99)
Mild liver disease	164	1.11 (0.94, 1.30)
Diabetes type I and II	818	1.10 (1.02, 1.18)
Hemiplegia	29	1.14 (0.78, 1.68)
Moderate to severe renal disease	159	1.25 (1.06, 1.48)
Diabetes with end organ damage	270	1.04 (0.91, 1.18)
Any tumor	1,135	1.17 (1.10, 1.25)
Leukemia	22	0.82 (0.53, 1.27)
Lymphoma	78	1.07 (0.85, 1.36)
Moderate to severe liver disease	39	1.86 (1.32, 2.62)
Metastatic solid tumor	75	1.49 (1.17, 1.89)
AIDS	1	0.05 (0.07, 3.73)

Abbreviations. OR: odds ratio.

**Table 4:** Original and updated Charlson Comorbidity Index (CCI) score, individual Charlson diseases, age groups and their association with distant breast cancer stage.

	Cases, N	OR (95% CI)^a^
Stage		
Local	21,576 (47)	
Regional	18,306 (40)	
Distant	3,058 (6.6)	
**Original CCI score** b		
0	37,539 (87)	ref
1	3,201 (7.4)	0.90 (0.79, 1.04)
2	1,601 (3.7)	0.85 (0.70, 1.03)
3	380 (0.9)	0.95 (0.66, 1.37)
≥4	219 (0.5)	0.97 (0.60, 1.58)
**Updated CCI score** b		
0	39,444 (92)	ref
1	1,656 (3.9)	0.93 (0.77, 1.22)
2	1,454 (3.4)	0.86 (0.70, 1.05)
3	215 (0.5)	1.20 (0.77, 1.87)
≥4	171 (0.4)	0.77 (0.41, 1.41)
**Charlson disease** b		
Myocardial infarction	373 (0.9)	0.68 (0.44, 1.04)
Congestive heart failure	410 (1.1)	0.79 (0.55, 1.15)
Peripheral vascular disease	466 (1.1)	1.00 (0.72, 1.38)
Cerebrovascular disease	942 (2.2)	0.78 (0.60, 1.00)
Dementia	69 (0.2)	1.03 (0.47, 2.27)
Chronic pulmonary disease	1,141 (2.7)	0.88 (0.70, 1.11)
Connective tissue disease	576 (1.3)	1.03 (0.76, 1.39)
Ulcer disease	468 (1.1)	1.05 (0.76, 1.46)
Mild liver disease	150 (0.4)	0.84 (0.43, 1.65)
Diabetes type I and II	733 (1.7)	0.94 (0.72, 1.24)
Hemiplegia	27 (0.06)	2.03 (0.70, 5.94)
Moderate to severe renal disease	141 (0.3)	1.24 (0.70, 2.21)
Diabetes with end organ damage	237 (0.6)	1.00 (0.63, 1.59)
Any tumor	1,031 (2.4)	0.90 (0.71, 1.14)
Leukemia^c^	21 (0.05)	1.22 (0.28, 5.28)
Lymphoma^c^	70 (0.2)	–
Moderate to severe liver disease	36 (0.08)	–
Metastatic solid tumor	66 (0.2)	1.64 (0.78, 3.46)
AIDS^c^	1 (0)	–
**Age group** d		
45–50	4,630 (11)	ref
51–60	12,651 (29)	1.18 (1.01, 1.39)
61–70	13,211 (31)	1.54 (1.32, 1.80)
71–80	9,050 (21)	2.26 (1.93, 2.64)
81–85	3,398 (7.9)	2.99 (2.51, 3.55)

a Calculated as distant stage vs. local/regional stage breast cancer at diagnosis. Patients with unknown stage (n = 3,384) were excluded from the analysis.

b Adjusted for age as a continuous variable.

c Zero cases of distant stage breast cancer.

d Adjusted for original CCI score in five categories.

Abbreviations. OR: odds ratio.

### Hypothesis-screening Analysis

In the hypothesis-screening analysis, hospital diagnoses recorded in the three years preceding the index date, representing 54.4% of all diagnoses, were excluded. After morbidity categories with fewer than five exposed cases and those affecting only men were excluded, 155 morbidity categories remained for analysis. Overall, ORs were skewed towards an increased risk of breast cancer for these 155 morbidity categories, with few ORs below the null. We obtained a pooled OR estimate of 1.07 (95% CI: 1.06, 1.08) associating any morbidity in the three to ten years preceding the index date with breast cancer risk. Of the 155 morbidity categories, iron deficiency anemia (OR = 0.61, 95% CI: 0.45, 0.81), other anemias (OR = 0.78, 95% CI: 0.66, 0.94), osteoporosis with and without fracture (OR = 0.87, 95% CI: 0.78, 0.96), rheumatoid arthritis and other inflammatory polyarthropathies (OR = 0.88, 95% CI: 0.80, 0.98), gastric and duodenal ulcer (OR = 0.89, 95% CI: 0.81, 0.98), and acute myocardial infarction (OR = 0.89, 95% CI: 0.81, 0.99) were inversely associated with subsequent breast cancer. Several morbidity categories, such as previous cancer diseases, were initially positively associated with breast cancer. After EB adjustment, however, ORs for only two diseases indicated a statistically significant association: disorders of the breast (EB−OR = 1.54, 95% CI: 1.28, 1.84) and other *in situ* and benign neoplasms and neoplasms of uncertain and unknown behavior (EB−OR = 1.30, 95% CI: 1.09, 1.55). The 20 associations most strongly negatively and positively associated with breast cancer are presented in Table 5 and Table 6, respectively. A complete list of the associations of the 155 morbidity categories with breast cancer and the corresponding original and EB- adjusted estimates are presented in Table S3.

**Table 5:** The 20 preceding morbidity categories most strongly associated with decreased breast cancer incidence.

Preceding morbidity category	Exposed cases, n	OR (95% CI)	EB-OR (95% CI)
Iron deficiency anemia	32	0.61 (0.45, 0.81)	0.91 (0.73, 1.15)
Chronic disease of tonsils and adenoids	59	0.74 (0.52, 1.06)	0.99 (0.78, 1.25)
Dementia	5	0.77 (0.59, 1.00)	0.96 (0.77, 1.20)
Malignant neoplasm of bone and articular cartilage	50	0.77 (0.31, 1.91)	1.05 (0.82, 1.33)
Other tuberculosis	10	0.78 (0.41, 1.48)	1.04 (0.81, 1.32)
Other anemias	133	0.78 (0.66, 0.94)	0.91 (0.74, 1.13)
Alzheimer’s disease	18	0.79 (0.49, 1.28)	1.02 (0.81, 1.30)
Other diseases of the bone	104	0.83 (0.68, 1.01)	0.95 (0.77, 1.19)
Other malignant neoplasms of urinary tract	16	0.85 (0.51, 1.41)	1.03 (0.81, 1.31)
Septicemia	53	0.85 (0.64, 1.12)	0.99 (0.79, 1.25)
Influenza	39	0.85 (0.61, 1.18)	1.01 (0.80, 1.27)
Hepatitis	19	0.86 (0.54, 1.37)	1.03 (0.81, 1.31)
Migraine	114	0.86 (0.71, 1.04)	0.96 (0.78, 1.19)
Osteoporosis with and without fracture	369	0.87 (0.78, 0.96)	0.92 (0.75, 1.11)
Rheumatoid arthritis and other inflammatory polyarthropathies	438	0.88 (0.80, 0.98)	0.92 (0.76, 1.12)
Gastric and duodenal ulcer	467	0.89 (0.81, 0.98)	0.93 (0.77, 1.12)
Acute myocaridal infarction	429	0.89 (0.81, 0.99)	0.93 (0.76, 1.13)
Benign neoplasm of brain and other parts of central nervous system	45	0.90 (0.67, 1.23)	1.02 (0.81, 1.28)
Epilepsy	185	0.91 (0.78, 1.05)	0.97 (0.79, 1.19)
Malignant neoplasm of larynx	47	0.91 (0.49, 1.70)	1.05 (0.82, 1.33)

Abbreviations. OR: odds ratio; EB-OR: Empirical-Bayes adjusted odds ratios.

**Table 6:** The 20 preceding morbidity categories most strongly associated with increased breast cancer incidence.

Preceding morbidity category	Exposed cases, n	OR (95% CI)	EB-OR (95% CI)
Conduction disorders and cardiac arrhythmias	998	1.23 (1.15, 1.31)	1.20 (1.00, 1.44)
Intracranial hemorrhage	114	1.23 (1.01, 1.50)	1.13 (0.91, 1.40)
Congestive heart failure	469	1.24 (1.12, 1.36)	1.19 (0.98, 1.44)
Delivery without mention of complication	217	1.24 (1.07, 1.43)	1.16 (0.95, 1.42)
Other intestinal infectious disease	79	1.26 (1.00, 1.60)	1.13 (0.90, 1.41)
Malignant neoplasm of colon	171	1.27 (1.08, 1.48)	1.17 (0.95, 1.44)
Mood affective disorders	36	1.31 (0.93, 1.85)	1.10 (0.88, 1.39)
Other *in situ* and benign neoplasms and neoplasms of uncertain and unknown behavior	2179	1.32 (1.26, 1.38)	1.30 (1.09, 1.55)
Female infertility	48	1.32 (0.98, 1.79)	1.12 (0.89, 1.40)
Malignant neoplasm of other, ill-defined, secondary, unspecified and multiple sites	69	1.33 (1.03, 1.70)	1.14 (0.91, 1.42)
Osteomyelitis and periostitis	27	1.37 (0.92, 2.05)	1.10 (0.87, 1.39)
Nephritis and nephrosis	44	1.38 (1.01, 1.89)	1.13 (0.89, 1.42)
Myostitis	15	1.39 (0.81, 2.38)	1.09 (0.86, 1.38)
Hodgkin’s disease	11	1.39 (0.74, 2.62)	1.08 (0.85, 1.37)
Schizophrenia, schizotypal, and delusional disorders	31	1.51 (1.04, 2.21)	1.13 (0.89, 1.42)
Malignant neoplasm of lip, oral cavity and pharynx	46	1.51 (1.11, 2.07)	1.15 (0.92, 1.45)
Other congenital malformations of the digestive system	26	1.56 (1.03, 2.35)	1.12 (0.89, 1.42)
Other malformations of the genitourinary system	49	1.62 (1.20, 2.19)	1.18 (0.94, 1.48)
Disorders of breast^a^	1052	1.62 (1.52, 1.73)	1.54 (1.28, 1.84)
Acute poliomyelitis	18	1.94 (1.17, 3.21)	1.13 (0.89, 1.43)

Abbreviations. OR: odds ratio; EB-OR: Empirical-Bayes adjusted odds ratios.

a Disorders of the breast include benign mammary dysplasia, inflammatory disorders of breast, hypertrophy of breast, unspecified lump in breast, and other disorders of breast.

## Discussion

In the present study, increasing CCI score calculated by either the original [20] or an updated [21] CCI score, and based on diagnoses three to ten years before the index date, were associated with subsequent risk of breast cancer. The distribution of odds ratios for 155 morbidity categories was skewed towards a causal association. After EB adjustment was applied to the estimates obtained in the hypothesis-screening analysis, however, only “disorders of the breast” and “other *in situ* and benign neoplasms and neoplasms of uncertain and unknown origin” remained clearly associated with breast cancer.

### Strengths and Limitations

The relatively homogeneous Danish population and Denmark’s tax-supported health care system provide an optimal setting for the conduct of population-based case-control studies. This nationwide study comprised all Danish women between 45 and 85 years of age diagnosed with breast cancer between 1994 and 2008 and their matched controls. Registration of breast cancer diagnoses in the DCR is nearly complete [26].

Study limitations include the completeness of diagnoses recorded in the NRP and several potential confounding factors. While the positive predictive values of the Charlson diseases in the NRP are high [32], many diseases and complications diagnosed in primary care are not recorded in the NRP. This under-registration could diminish potential effects of specific conditions.

Misclassification of diseases, such as those resulting from changes in clinical interpretations of ICD-codes [33], could also bias associations. In the hypothesis-screening analysis, some morbidity categories represented combinations of ICD-codes for medical conditions with different etiologies, possibly leading to bias. Another concern is that we excluded all diagnoses between the index date and the three preceding years, and this presumed latency period may not be appropriate for all diseases. However, including all diagnoses recorded in the NRP preceding the index date, with or without a three year latency period, did not change the results notably (data not shown).

We were unable to control for confounding by differential treatment of diseases and complications, genetic markers, reproductive history, oral contraceptives, hormone replacement therapy, lifestyle, or region of residence, and many of those factor could themselves explain the increased or decreased risk of breast cancer associated with diseases. Given the matching criteria, which included birth year, lack of information on menopausal status is unlikely to have had a major impact on our findings. Results were stratified by age groups corresponding to typical categories of pre-menopausal, peri-menopausal, and post-menopausal women.

The association between increasing CCI score of up to six and breast cancer could be related to close medical surveillance of patients burdened with many medical conditions. However, it is also possible that physicians caring for patients with high numbers of diseases may tend to focus on these diseases and neglect to search for non-symptomatic conditions, such as breast cancer. Increasing CCI score and presence of individual Charlson diseases were associated with elevated proportions of distant stage breast cancer; however, with logistic regression models that adjusted for age, there was no association between the original or updated CCI score or individual Charlson diseases and distant stage breast cancer at diagnosis.

The updated CCI included only the 14 diseases from the original CCI, with “any tumor”, “leukemia” and “lymphoma” combined with a weight of 2 [21]. In the current study, the latter diseases were each assigned a weight of 2, both with the original and the updated CCI versions. This approach did not noticeably affect our estimates (data not shown). When we excluded any cancer disease from the CCI, resulting in decreased overall CCI score, the risk of breast cancer associated with scores of up to six was reduced for the original index but not for the updated index (data not shown). It may be, therefore, that cancer diseases drive much of the observed association, for example through shared risk factors between breast cancer and other cancer diseases or as a side effect of treatment for the original cancer. Moreover, many cancers and many other Charlson diseases are associated with lifestyle factors, such as smoking and alcohol consumption [34]. Renal failure is a serious side effect of adjuvant chemotherapy [35], and diabetes can be induced by glucocorticoids or physical inactivity [36].

The associations found between many of the individual 155 morbidity categories and breast cancer is not surprising (Table 5 and 6 and Table S3). Some associations may relate to established risk factors, such as cumulative estrogen exposure, genetics, or lifestyle. For example, “disorders of breast” was strongly associated with breast cancer even after EB adjustment. However, this morbidity category consists of several diseases (see notes under Table 6), some of which are not established breast cancer risk factors. Osteoporosis, heart disease, gastric ulcer, and rheumatoid arthritis, were initially inversely associated with breast cancer. Acetylsalicylic acid (aspirin) is used to treat rheumatoid arthritis, osteoporosis and heart disease, and gastric ulcer and bleeding and thus anemia are well known complications to regular aspirin intake. A recent review and meta-analysis concluded that aspirin reduces the risk of breast cancer [19], so these associations may reflect a protective effect of aspirin associated with the preceding morbidities. Anemia was also associated with a decreased risk of breast cancer before EB adjustment, and recent studies suggest that excess endogenous iron storage raises the risk of breast cancer [37], [38]. It is possible, therefore, that the negative association observed with anemia could be mediated by changes in iron homeostasis.

### Conclusions

In conclusion, our study does not provide support for any substantial association between morbidity measured with the original and an updated CCI and breast cancer risk. The hypothesis-screening analysis suggests novel associations that may merit further attention, such as potential protective effects of acetylsalicylic acid or iron deficiency.

## Supporting Information

Table S1 Specification of Charlson diseases, ICD-8 and ICD-10 codes, and the original and updated Charlson morbidity index score weights.
(DOC)

Table S2 List of 202 disease categories and associated ICD-8 and ICD-10 codes.
(DOC)

Table S3 Morbidity categories preceding breast cancer diagnosis, number of exposed cases, corresponding odds ratios (ORs) and Empirical-Bayes-adjusted estimates accompanied by 95% CI for associations between morbidity categories and breast cancer incidence.
(DOC)
