# Supplementary material for: Hospital Recorded Morbidity and Breast Cancer Incidence: A Nationwide Population-Based Case-Control Study
Source: PLoS One. 2012 Oct 19;7(10):e47329. doi: 10.1371/journal.pone.0047329 (PMC3477157; doi:10.1371/journal.pone.0047329)
Supplement: Table S1 — Specification of Charlson diseases, ICD-8 and ICD-10 codes, and the original and updated Charlson morbidity index score weights. (DOC) [file pone.0047329.s001.doc]

| **Supporting Information Table 1: Specification of Charlson diseases, ICD-8 and ICD-10 codes, and the original Charlson, and updated Charlson comorbidity index score weights.** | | | | |
| --- | --- | --- | --- | --- |
| **Charlson comorbidity index variable** | **ICD-8** | **ICD-10** | **Charlson weight** | **Updated weight** |
| Myocardial infarction | 410 | I21, I22, I23 | 1 | 0 |
| Congestive heart failure | 427.09, 427.10, 427.11, 427.19, 428.99, 782.49 | I50, I11.0, I13.0, I13.2 | 1 | 2 |
| Peripheral vascular disease | 440, 441, 442, 443, 444, 445 | I70, I71, I72, I73, I74, I77 | 1 | 0 |
| Cerebrovascular disease | 430-438 | I60-I69, G45, G46 | 1 | 0 |
| Dementia | 290.09-290.19, 293.09 | F00-F03, F05.1, G30 | 1 | 2 |
| Chronic pulmonary disease | 490-493, 515-518 | J40-J47, J60-J67, J68.4, J70.1,  J70.3, J84.1, J92.0, J96.1, J98.2, J98.3 | 1 | 1 |
| Connective tissue disease | 712, 716, 734, 446, 135.99 | M05, M06, M08, M09,M30,M31, M32, M33, M34, M35, M36, D86 | 1 | 1 |
| Ulcer disease | 530.91, 530.98, 531-534 | K22.1, K25-K28 | 1 | 0 |
| Mild liver disease | 571, 573.01, 573.04 | B18, K70.0-K70.3, K70.9, K71, K73, K74, K76.0 | 1 | 2 |
| Diabetes type1  Diabetes type2 | 249.00,249.06, 249.07, 249.09  250.00,250.06, 250.07, 250.09 | E10.0, E10.1, E10.9  E11.0, E11.1, E11.9 | 1 | 0 |
| Hemiplegia | 344 | G81, G82 | 2 | 2 |
| Moderate to severe renal disease | 403, 404, 580-583,584,590.09, 593.19, 753.10-753.19, 792 | I12, I13, N00-N05, N07, N11, N14, N17-^N19^, Q61 | 2 | 1 |
| Diabetes with end organ damage type1 type2 | 249.01-249.05, 249.08 250.01-250.05, 250.08 | E10.2-E10.8 E11.2-E11.8 | 2 | 1 |
| Any tumor | 140-194 | C00-C75 | 2 | 2 |
| Leukemia | 204-207 | C91-C95 | 2 | 2 |
| Lymphoma | 200-203,275.59 | C81-C85, C88, C90, C96 | 2 | 2 |
| Moderate to severe liver disease | 070.00, 070.02, 070.04, 070.06, 070.08, 573.00, 456.00-456.09 | B15.0, B16.0, B16.2, B19.0, K70.4, K72, K76.6, I85 | 3 | 4 |
| Metastatic solid tumor | 195-198, 199 | C76-C80 | 6 | 6 |
| AIDS | 079.83 | B21-B24 | 6 | 4 |
