# Supplementary material for: Hospital Recorded Morbidity and Breast Cancer Incidence: A Nationwide Population-Based Case-Control Study
Source: PLoS One. 2012 Oct 19;7(10):e47329. doi: 10.1371/journal.pone.0047329 (PMC3477157; doi:10.1371/journal.pone.0047329)
Supplement: Table S2 — List of 202 disease categories and associated ICD-8 and ICD-10 codes. (DOC) [file pone.0047329.s002.doc]

| **Supporting Information Table 2: List of 202 disease categories and associated ICD-8 and ICD-10 codes** | |
| --- | --- |
| **Disease category^a^** | **ICD codes** |
| Cholera | ICD-8: 0.0–0.9 ICD-10: A00.0–A00.9 |
| Typhoid and paratyphoid fevers | ICD-8: 1.0–1.9  ICD-10: A01.0–A01.9 |
| Other intestinal infectious diseases | ICD-8: 5.0–5.9 ICD–8: 7.0-7.9 ICD-10: A02.0–A02.9  ICD-10: A04.0–A05.9  ICD-10: A07.0–A08.9 |
| Shigellosis/Bacillary dysentery | ICD-8: 4.0–4.9  ICD-10: A03.0–A03.9 |
| Amoebiasis | ICD-8: 6.0–6.9 ICD-10: A06.0–A06.9 |
| Diarrhea and gastro-enteritis of presumed infectious origin | ICD-8: 8.0–9.9 ICD-10: A09.0–A09.9 |
| Respiratory tuberculosis | ICD-8: 10.0–12.3 ICD-10: A15.0–A16.9 |
| Other tuberculosis | ICD-8: 13.0–19.9 ICD-10: A17.0–A19.9  ICD-10: B90.0–B90.9 |
| Plague | ICD-8: 20.0–20.9 ICD-10: A20.0–A20.9 |
| Other bacterial diseases | ICD-8:21.0–22.9 ICD-8: 24.0–27.9 ICD-8: 31.0–31.9  ICD-8: 34.0–34.1 ICD-8: 35.0–35.9  ICD-8: 30.0–39.9 ICD-10: A21.0–A22.9  ICD-10: A24.0–A28.9  ICD-10: A31.0–A32.9 ICD-10: A38.0–A38.9  ICD-10: A42.0–A49.9  ICD-10: B96.0–B96.9 |
| Brucellosis | ICD-8: 23.0–23.9 ICD-10: A23.0–A23.9 |
| Leprosy | ICD-8:30.0–30.9 ICD-10: A30.0–A30.9 ICD-10: B92.0–B92.9 |
| Tetanus | ICD-8: 37.0–37.9 ICD-10: A33.0–A33.9  ICD-10: A34.0–A35.9 |
| Diphtheria | ICD-8: 32.0–32.9 ICD-10: A36.0–A36.9 |
| Whooping cough | ICD-8: 33.0–33.9 ICD-10: A37.0–A37.9 |
| Meningococcal infection | ICD-8: 36.0–36.9 ICD-10: A39.0–A39.9 |
| Septicemia | ICD-8: 38.0–38.9 ICD-10: A40.0–A41.9 |
| Early syphilis | ICD-8: 91.0–91.9  ICD-10: A51.0–A51.9 |
| Other syphilis | ICD-8: 90.0–90.9 ICD-8: 92.0–97.9 ICD-10: A50.0–A50.9  ICD-10: A52.0–A53.9 |
| Gonococcal infection | ICD-8: 98.0–98.9 ICD-10: A54.0–A54.9 |
| Other infectious and parasitic diseases | ICD-8: 130.0–136.9  ICD-8: 89.0–89.9  ICD-8: 99.0–117.9 ICD-10: A55.0–A67.9  ICD-10: A69.0–A70.9  ICD-10: A74.0–A74.9  ICD-10: A77.0–A79.9  ICD-10: B35.0–B49.9  ICD-10: B58.0–B64.9  ICD-10: B85.0–B89.9  ICD-10: B94.0–B94.9 ICD-10: B99.0–B99.9 |
| Relapsing fevers | ICD-8: 88.0–88.9 ICD-10: A68.0–A68.9 |
| Other viral diseases | ICD-8: 45.0–46.9 ICD-8: 50.0–54.9  ICD-8: 57.0–57.9  ICD-8: 61.0–61.9  ICD-8: 66.0–66.9  ICD-8: 68.0–68.9  ICD-8: 73.0–79.9 ICD-10: A71.0–A71.9 ICD-10: A81.0–A81.9  ICD-10: A87.0–A89.9  ICD-10: B00.0–B04.9  ICD-10: B07.0–B09.9  ICD-10: B20.0–B25.9  ICD-10: B27.0–B34.9  ICD-10: B97.0–B97.9 |
| Typhus and other rickettsioses | ICD-8: 80.0–83.9 ICD-10: A75.0–A75.9 |
| Acute poliomyelitis | ICD-8: 40.0–44.9 ICD-10: A80.0–A80.9  ICD-10: B91.0–B91.9 |
| Rabies | ICD-8: 71.0–71.9 ICD-10: A82.0–A82.9 |
| Viral encephalitis | ICD-8: 62.0–65.9 ICD-10: A83.0–A86.9 |
| Other arthropod-borne viral fevers and viral hemorrhagic fevers | ICD-8: 67.0–67.9 ICD-10: A90.0–A94.9 ICD-10: A96.0–A99.9 |
| Yellow fever | ICD-8: 60.0–60.9 ICD-10: A95.0–A95.9 |
| Measles | ICD-8: 55.0–55.9 ICD-10: B05.0–B05.9 |
| Rubella | ICD-8: 56.0–56.9  ICD-10: B06.0–B06.9 |
| Hepatitis | ICD-8: 70.0–70.9 ICD-10: B15.0–B15.9  ICD-10: B16.0–B16.9  ICD-10: B17.0–B19.9 |
| Mumps | ICD-8: 72.0–72.9 ICD-10: B26.0–B26.9 |
| Malaria | ICD-8: 84.0–84.9 I CD-10: B50.0–B54.9 |
| Leishmaniasis | ICD-8: 85.0–85.9  ICD-10: B55.0–B55.9 |
| Trypanosomiasis | ICD-8: 86.0–87.9  ICD-10: B56.0–B57.9 |
| Schistosomiasis | ICD-8: 120.0–120.9 ICD-10: B65.0–B65.9 |
| Other helminthiase | ICD-8: 121.0–121.9  ICD-8: 123.0–125.9  ICD-8: 127.0–129.9 ICD-10: B66.0–B66.9  ICD-10: B68.0–B75.9 ICD-10: B77.0–B83.9 |
| Echinococcosis/hydatidosis | ICD-8: 122.0–122.9  ICD-10: B67.0–B67.9 |
| Hookworm diseases/Ankylostomiasis | ICD-8: 126–126.9 ICD-10: B76.0–B76.9 |
| Malignant neoplasm of lip, oral cavity and pharynx | ICD-8: 140.0–149.9 ICD-10: C00.0–C14.9 |
| Malignant neoplasm of other digestive organs and peritoneum | ICD-8: 150.0–150.9 ICD-8: 155.0–159.9 ICD-10: C15.0–C15.9  ICD-10: C17.0–C17.9  ICD-10: C22.0–C26.9 |
| Malignant neoplasm of stomach | ICD-8: 151.0–151.9 ICD-10: C16.0–C16.9 |
| Malignant neoplasm of colon | ICD-8: 152.0–153.9 ICD-10: C18.0–C18.9 |
| Malignant neoplasm of rectosigmoid junction, rectum, anus, and anal canal | ICD-8: 154.0–154.9 ICD-10: C19.0–C21.9 |
| Other malignant neoplasms of respiratory and intrathoracic organs | ICD-10: C30.0–C31.9  ICD-10: C37.0–C39.9 |
| Malignant neoplasm of other and unspecified respiratory organs | ICD-8: 160.0–160.9 ICD-8: 163.0–163.9 |
| Malignant neoplasm of larynx | ICD-8: 161.0–161.9 ICD-10:C32.0–C32.9 |
| Malignant neoplasm of trachea, bronchus and lung | ICD-8: 162.0–162.9 ICD-10: C33.0–C34.9 |
| Malignant neoplasm of bone and articular cartilage | ICD-8: 170.0–170.9 ICD-10: C40.0–C41.9 |
| Malignant neoplasm of skin | ICD-8: 172.0–173.9 ICD-10: C43.0–C43.9  ICD-10: C44.0–C44.9 |
| Malignant neoplasm of other specified sites | ICD-8: 171.0–171.9  ICD-8: 190.0–190.9  ICD-8: 192.0–195.9 ICD-10: C45.0–C49.9  ICD-10: C69.0–C70.9  ICD-10: C72.0–C72.9 |
| Other malignant neoplasms of female genital organs | ICD-8: 181.0–181.9 ICD-8: 183.0–183.1 ICD-8: 183.0–184.9 ICD-10: C51.0–C52.9  ICD-10: C56.0–C58.9 |
| Malignant neoplasm of *cervix uteri* | ICD-8: 180.0–180.9 ICD-10: C53.0–C53.9 |
| Malignant neoplasm of other and unspecified parts of uterus | ICD-8: 182.0–182.9  ICD-10: C54.0–C55.9 |
| Other malignant neoplasms of male genital organs | ICD-8: 186.0–186.9 ICD-10: C60.0–C60.9  ICD-10: C62.0–C63.9 |
| Malignant neoplasm of prostate | ICD-8: 185.0–185.9 ICD-10: C61.0–C61.9 |
| Other malignant neoplasms of urinary tract | ICD-10: C64.0–C66.9  ICD-10: C68.0–C68.9 |
| Malignant neoplasm of other genitourinary organs | ICD-8: 187.0–187.9  ICD-8: 189.0–189.9 |
| Malignant neoplasm of bladder | ICD-8: 188.0–188.9 ICD-10: C67.0–C67.9 |
| Malignant neoplasm of brain | ICD-8: 191.0–191.9 ICD-10: C71.0–C71.9 |
| Malignant neoplasm of other, ill-defined, secondary, unspecified,  and multiple sites | ICD-8: 197.0–199.9  ICD-10: C73.0–C80. ICD-10: C97.0–C97.9 |
| Hodgkin’s disease | ICD-8: 201.0–201.9  ICD-10: C81.0–C81.9 |
| Other malignant neoplasms of lymphoid, hematopoietic, and related tissue | ICD-8:196.0–196.9 ICD-8: 200.0–200.9 ICD-8: 202.0–203.9 ICD-8: 208.0–209.9 ICD-10: C82.0–C85.9  ICD-10: C88.0–C90.9  ICD-10: C96.0–C96.9 |
| Leukemia | ICD-8: 204.0–207.9  ICD-10: C91.0–C95.9 |
| Other *in situ* and benign neoplasms and neoplasms of uncertain and unknown behavior | ICD-8: 210.0–215.9 ICD-8: 217.0–217.9 ICD-8: 219.0–219.9 ICD-8: 221.0–222.9 ICD-8: 224.0–224.9 ICD-8: 226.0–228.9 ICD-8: 230.0–239.9 ICD-10: D00.0–D05.9 ICD-10: D07.0–D21.9 ICD-10: D24.0–D24.9 ICD-10: D26.0–D26.9 ICD-10: D28.0–D29.9 ICD-10: D31.0–D32.9 ICD-10: D34.0–D48.9 |
| Carcinoma *in situ* of *cervix uteri* | ICD-8: 234.0–234.0 ICD-10: D06.0–D06.9 |
| Benign neoplasm of skin | ICD-8: 216.0–216.9 ICD-10: D22.0–D23.9 |
| Leiomyoma of uterus | ICD-8: 218.0–218.9 ICD-10: D25.0–D25.9 |
| Benign neoplasm of ovary | ICD-8: 220.0–220.9 ICD-10: D27.0–D27.9 |
| Benign neoplasm of kidney and other urinary organs | ICD-8: 223.0–223.9 ICD-10: D30.0–D30.9 |
| Benign neoplasm of brain and other parts of central nervous system | ICD-8: 225.0–225.9 ICD-10: D33.0–D33.9 |
| Iron deficiency anemia | ICD-8: 280.0–280.9 ICD-10: D50.0–D50.9 |
| Other anemias | ICD-8: 281.0–285.9 ICD-10: D51.0–D64.9 |
| Hemorrhagic conditions and other diseases of blood and blood-forming organs | ICD-8:286.0–289.9 ICD-10: D65.0–D77.9 |
| Other endocrine, nutritional. and metabolic disorders | ICD-8: 251.0–258.9  ICD-8: 270.0–279.9 ICD-10: D80.0–D89.9  ICD-10: E15.0–E35.9  ICD-10: E58.0–E63.9  ICD-10: E65.0–E65.9  ICD-10: E66.0–E66.9  ICD-10: E67.0–E85.9 ICD-10: E87.0–E90.9 |
| Other disorders of thyroid | ICD-8: 240.0–241.9  ICD-8: 243.0–246.9 ICD-10: E03.0–E04.9  ICD-10: E06.0–E07.9 |
| Iodine-deficiency-related thyroid disorders | ICD-8: 242.0–242.9 ICD-10: E00.0–E02.9  ICD-10: E05.0–E05.9 |
| Diabetes mellitus | ICD-8: 249.0–250.9 ICD-10: E10.0–E14.9 |
| A-vitaminosis and other nutritional deficiency | ICD-8: 260.0–269.9 ICD-10: E40.0–E47.9 ICD-10:E50.0–E50.9 ICD-10: E51.0-E56.9 ICD-10: E64.0–E64.9 |
| Dementia | ICD-8: 290.0–290.0  ICD-8: 290.0–290.9 ICD-10: F00.0–F03.9  ICD-10: G31.0–G31.0 |
| Other mental and behavioral disorders | ICD-8: 292.0–294.9  ICD-8: 297.0–299.9  ICD-8: 305.0–309.9 ICD-10: F04.0–F09.9  ICD-10: F50.0–F69.9  ICD-10: F80.0–F99.9 |
| Alcohol-, drug-abuse-related disease | ICD-8: 291.0–291.9  ICD-8: 303.0–304.9  ICD-10: F10.0–F19.9 |
| Schizophrenia, schizotypal, and delusional disorders | ICD-8: 295.0–295.9 ICD-10: F20.0–F29.9 |
| Mood (affective) disorders | ICD-8: 296.0–296.1  ICD-8: 296.0–296.9  ICD-10: F30.0–F31.9 ICD-10: F34.0–F39.9 |
| Depression | ICD-8: 296.0–296.0  ICD-8: 296.0–296.2 ICD-10: F32.0–F33.9 |
| Neurotic, stress-related, and somatoform disorders | ICD-8: 300.0–302.9 ICD-10: F40.0–F48.9 |
| Mental retardation | ICD-8: 310.0–315.9 ICD-10: F70.0–F79.9 |
| Inflammatory diseases of the central nervous system | ICD-8: 320.0–320.9 ICD-8: 321.0–324.9  ICD-10: G00.0–G09.9 |
| Other diseases of the nervous system | ICD-8: 330.0–333.9 ICD-8: 343.0–344.9 ICD-8: 347.0–358.9 ICD-10: G10.0–G13.9 ICD-10: G21.0–G26.9 ICD-10: G31.1–G32.9 ICD-10: G36.0–G37.9 ICD-10: G44.0–G44.9 ICD-10: G46.0–G47.9 ICD-10:G50.0–G73.9 ICD-10: G80.0–G83.9 ICD-10: G90.0–G99.9 |
| Parkinson’s disease | ICD-8: 342.0–342.9 ICD-10: G20.0–G20.9 |
| Alzheimer’s disease | ICD-8: 290.0–290.1  ICD-10: G30.0–G30.9 |
| Multiple sclerosis and other demyelinating diseases | ICD-8: 340.0–341.9 ICD-10: G35.0–G35.9 |
| Epilepsy | ICD-8: 345.0–345.9 ICD-10: G40.0–G41.9 |
| Migraine | ICD-8: 346.0–346.9 ICD-10: G43.0–G43.9 |
| Transient cerebral ischemic attacks and related syndromes | ICD-8: 435.0–435.9 ICD-10: G45.0–G45.9 |
| Other inflammatory diseases of eye | ICD-8: 360.0–369.9 ICD-10: H00.0–H01.9  ICD-10: H10.0–H13.9  ICD-10: H15.0–H19.9 |
| Other diseases of the eye and adnexa | ICD-8: 370.0–372.9  ICD-8: 377.0–379.9 ICD-10: H02.0–H06.9  ICD-10: H20.0–H22.9  ICD-10: H30.0–H32.9  ICD-10: H34.0–H36.9  ICD-10: H43.0–H48.9  ICD-10: H51.0–H59.9 |
| Cataract and other disorders of lens | ICD-8: 374.0–374.9 ICD-10: H25.0–H28.9 |
| Retinal detachments and breaks | ICD-8: 376.0–376.9 ICD-10: H33.0–H33.9 |
| Glaucoma | ICD-8: 375.0–375.9 ICD-10: H40.0–H42.9 |
| Strabismus | ICD-8: 373.0–373.9 ICD-10: H49.0–H50.9 |
| Other diseases of the ear and mastoid process | ICD-8: 380.0–380.9 ICD-8: 381.0–381.9  ICD-8: 382.0–383.9 ICD-8: 384.0–389.9 ICD-10: H60.0–H62.9 ICD-10: H65.0–H75.9  ICD-10: H80.0–H83.9  ICD-10: H90.0–H95.9 |
| Acute rheumatic fever | ICD-8: 390.0–392.9 ICD-10: I00.0–I02.9 |
| Chronic rheumatic heart disease | ICD-8: 393.0–392.2 ICD-10: I05.0–I09.9 |
| Essential (primary) hypertension | ICD-8: 400.0–404.9 ICD-10: I10.0–I15.9 |
| Angina pectoris | ICD-8: 413.0–413.9 ICD-10: I20.0–I20.9 |
| Acute myocardial infarction | ICD-8: 410.0–410.9 ICD-10: I21.0–I22.9 |
| Other ischemic heart diseases | ICD-8: 411.0–412.9  ICD-10: I23.0–I25.9 |
| Other ischemic heart disease | ICD-8: 414.0–414.9 |
| Pulmonary embolism | ICD-8: 450.0–450.9 ICD-10: I26.0–I26.9 |
| Other heart diseases | ICD-8: 420.0–426.9  ICD-8: 428.0–429.9 ICD-10: I27.0–I43.9  ICD-10: I51.0–I52.9 |
| Conduction disorders and cardiac arrhythmias | ICD-8: 427.0–427.9 ICD-10: I44.0–I49.9 |
| Congestive heart failure | ICD-8: 427.0–427.0 ICD-10: I50.0–I50.9 |
| Intracranial hemorrhage | ICD-8: 431.0–431.9 ICD-10: I60.0–I62.9 |
| Cerebral infarction | ICD-8: 432.0–434.9 ICD-10: I63.0–I63.9 |
| Other cerebrovascular diseases | ICD-8: 430.0–430.9 ICD-8: 436.0–436.9 ICD-8: 437.0–438.9 ICD-10: I64.0–I64.9 ICD-10: I65.0–I69.9 |
| Atherosclerosis | ICD-8: 440.0–440.9 ICD-10: I70.0–I70.9 |
| Other diseases of arteries, arterioles and capillaries | ICD-8: 441.0–442.9  ICD-8: 444.0–448.9  ICD-10: I71.0–I72.9 ICD-10: I74.0–I74.9  ICD-10:I77.0–I79.9 |
| Other peripheral vascular diseases | ICD-8: 443.0–443.9 ICD-10: I73.0–I73.9 |
| Phlebitis, thrombophlebitis, venous embolism and thrombosis | ICD-8: 451.0–453.9 ICD-10: I80.0–I82.9 |
| Varicose veins of lower extremities | ICD-8: 454.0–454.9  ICD-10: I83.0–I83.9 |
| Hemorrhoids | ICD-8: 455.0–455.9 ICD-10: I84.0–I84.9 |
| Other diseases of the circulatory system | ICD-8: 456.0–458.9 ICD-10: I85.0–I99.9 |
| Other acute upper respiratory infections | ICD-8: 460–461.9 ICD-8: 464.0–465.9 ICD-10: J00.0–J01.9  ICD-10: J04.0–J04.9 ICD-10: J05.0–J06.9 |
| Acute pharyngitis and acute tonsillitis | ICD-8:34.0–34.0 ICD-8: 462.0–463.9 ICD-10: J02.0–J03.9 |
| Influenza | ICD-8: 470.0–474.9 ICD-10: J10.0–J11.9 |
| Pneumonia | ICD-8: 480.0–480.9 ICD-8: 481.0–481.9  ICD-8: 482.0–483.9  ICD-8: 484.0–486.9  ICD-10: J12.0–J18.9 |
| Acute bronchitis and acute bronchiolitis | ICD-8: 466.0–466.9 ICD-10: J20.0–J21.9 |
| Other diseases of the respiratory system | ICD-8: 510.0–514.9  ICD-8: 517.0–517.9  ICD-8: 519.0–519.9 ICD-10: J22.0–J22.9  ICD-10: J66.0–J99.9 |
| Other diseases of upper respiratory tract | ICD-8: 501.0–502.9  ICD-8: 504.0–504.9  ICD-8: 505.0–508.9 ICD-10: J30.0–J31.9 ICD-10: J33.0–J34.9  ICD-10: J36.0–J39.9 |
| Chronic sinusitis | ICD-8: 503.0–503.9 ICD-10: J32.0–J32.9 |
| Chronic disease of tonsils and adenoids | ICD-8: 500.0–500.9 ICD-10: J35.0–J35.9 |
| Bronchitis, emphysema and other chronic pulmonary diseases | ICD-8: 490.0–493.9  ICD-10: J40.0–J44.9 ICD-10: J45.0–J46.9 |
| Bronchiectasis | ICD-8: 518.0–518.9 ICD-10: J47.0–J47.9 |
| Pneumoconioses and related diseases | ICD-8: 515.0–516.9 ICD-10: J60.0–J65.9 |
| Other diseases of the teeth, oral cavity, salivary glands and jaws | ICD-8: 520.0–529.9  ICD-10: K00.0–K14.9 |
| Other diseases of esophagus, stomach and duodenum | ICD-8: 530.0–530.9 iCD-8: 536.0–537.9  ICD-10: K20.0–K23.9  ICD-10: K28.0–K28.9 ICD-10: K30.0–K31.9 |
| Gastric and duodenal ulcer | ICD-8: 531.0–534.9 ICD-10: K25.0–K27.9 |
| Gastritis and duodenitis | ICD-8: 535.0–535.9  ICD-10: K29.0–K29.9 |
| Diseases of appendix | ICD-8: 540.0–543.9  ICD-10: K35.0–K38.9 |
| Hernia | ICD-8: 550.0–553.9 ICD-10: K40.0–K46.9 |
| Crohn’s disease and ulcerative colitis | ICD-8: 563.0–563.9 ICD-10: K50.0–K51.9 |
| Other diseases of the digestive system | ICD-8: 561.0–562.9  ICD-8: 564.0–569.9 ICD-10: K52.0–K55.9  ICD-10: K57.0–K67.9  ICD-10: K82.0–K83.9  ICD-10: K87.0–K93.9 |
| Paralytic ileus and intestinal obstruction without hernia | ICD-8: 560.0–560.9  ICD-10: K56.0–K56.9 |
| Other diseases of liver and gallbladder | ICD-8: 570.0–573.9  ICD-8: 576.0–576.9  ICD-10: K70.0–K77.9 |
| Cholelithiasis and cholecystitis | ICD-8: 574.0–575.9 ICD-10: K80.0–K81.9 |
| Acute pancreatitis and other diseases of the pancreas | ICD-8:577.0–577.9 ICD-10: K85.0–K86.9 |
| Infections of the skin and subcutaneous tissue | ICD-8: 680.0–686.9 ICD-10: L00.0–L08.9 |
| Other diseases of the skin and subcutaneous tissue | ICD-8:690.0–698.9 ICD-8: 700.0–709.9  ICD-10: L10.0–L99.9 |
| Other disorders of joints | ICD-8: 724.0–724.9 ICD-8: 726.0–727.9 ICD-8:729.0–729.9 ICD-8:737.0–737.9 ICD-10: M00.0–M03.9 ICD-10: M22.0–M25.9 |
| Rheumatoid arthritis and other inflammatory polyarthropathies | ICD-8: 712.0–712.9  ICD-8: 716.0–716.9 ICD-10: M05.0–M14.9 |
| Osteoarthritis and allied conditions | ICD-8: 710.0–711.9 ICD-8: 713.0–715.9 ICD-10: M15.0–M19.9 ICD-10: M47.0–M47.9  ICD-10: M48.3–M48.3 |
| Acquired deformities of limbs | ICD-8: 736.0–736.9 ICD-10: M20.0–M21.9 |
| Other diseases of the musculoskeletal system and connective tissue | ICD-8: 730.0–730.9  ICD-8: 733.0–734.9  ICD-8: 738.0–738.9 ICD-10: M30.0–M36.9  ICD-10: M87.0–M90.9  ICD-10: M94.0–M99.9 |
| Other dorsopathies | ICD-8: 735.0–735.9 ICD-10: M40.0–M41.9 ICD-10: M43.0–M43.5 ICD-10: M43.7-M46.9  ICD-10: M48.0–M48.2  ICD-10: M48.4–M49.9 ICD-10: M53.0–M53.9 |
| Osteochondrosis | ICD-8: 722.0–722.9 ICD-10: M42.0–M42.9  ICD-10: M91.0–M93.9 |
| Rheumatism | ICD-8: 717.0–718.9  ICD-10: M43.6–M43.6  ICD-10: M79.0–M79.1 |
| Cervical and other intervertebral disc disorders | ICD-8: 725.0–725.9  ICD-8: 728.0–728.9 ICD-10: M50.0–M51.9 ICD-10: M54.0–M54.9 |
| Myositis | ICD-8: 732.0–732.9 ICD-10: M60.0–M60.9 |
| Soft tissue disorders | ICD-8: 731.0–731.9 ICD-10: M61.0–M78.9  ICD-10: M79.2–M79.5  ICD-10: M79.7–M79.9 |
| Osteoporosis with and without fracture | ICD-8: 723.0–723.0 ICD-10: M80.0–M81.9 |
| Other diseases of bone | ICD-8: 721.0–721.9  ICD-8: 723.0–723.9 ICD-10: M82.0–M85.9 |
| Osteomyelitis and periostitis | ICD-8: 720.0–720.9  ICD-10: M86.0–M86.9 |
| Nephritis and nephrosis | ICD-8: 580.0–584.9 ICD-10: N00.0–N08.9 |
| Infections of kidney | ICD-8: 590.0–590.9 ICD-10: N10.0–N16.9 |
| Other diseases of the urinary system | ICD-8: 591.0–591.9 ICD-8: 593.0–593.9 ICD-8:596.0–599.9 ICD-10: N17.0–N19.9  ICD-10: N25.0–N29.9  ICD-10: N31.0–N39.9 |
| Urolithiasis/Calculus of urinary system | ICD-8: 592.0–592.9  ICD-8: 594.0–594.9 ICD-10: N20.0–N23.9 |
| Cystitis | ICD-8: 595.0–595.9 ICD-10: N30.0–N30.9 |
| Hyperplasia of prostate | ICD-8: 600.0–600.9 ICD-10: N40.0–N40.9 |
| Other diseases of male genital organs | ICD-8: 601.0–602.9  ICD-8: 604.0–604.9  ICD-8: 606.0–607.9  ICD-10: N41.0–N42.9 ICD-10: N44.0–N46.9  ICD-10: N48.0–N51.9 |
| Hydrocele and spermatocele | ICD-8: 603.0–603.9 ICD-10: N43.0–N43.9 |
| Redundant prepuce, phimosis and paraphimosis | ICD-8: 605.0–605.9 ICD-10: N47.0–N47.9 |
| Disorders of breast | ICD-8: 610.0–611.9 ICD-10: N60.0–N64.9 |
| Salpingitis and oophoritis | ICD-8: 612.0–614.9 ICD-10: N70.0–N70.9 |
| Other inflammatory diseases of female pelvic organs | ICD-8: 622.0–622.9 ICD-10:N71.0–N71.9  ICD-10: N73.0–N77.9 |
| Inflammatory disease of cervix uteri | ICD-8: 620.0–620.9 ICD-10: N72.0–N72.9 |
| Endometriosis | ICD-8: 625.0–625.3 ICD-10: N80.0–N80.9 |
| Female genital prolapse | ICD-8: 623.0–623.9 ICD-10: N81.0–N81.9 |
| Other disorders of genitourinary tract | ICD-8:621.0–621.9 ICD-8: 624.0–625.9 ICD-8: 627.0–627.9  ICD-8: 629.0–629.9  ICD-10: N82.0–N82.9 ICD-10: N84.0–N90.9 ICD-10: N93.0–N96.9 ICD-10: N98.0–N99.9 |
| Other diseases of ovary, fallopian tube and parametrium | ICD-8: 615.0–616.9 ICD-10: N83.0–N83.9 |
| Disorders of menstruation | ICD-8: 626.0–626.9 ICD-10: N91.0–N92.9 |
| Female infertility | ICD-8: 628.0–628.9 ICD-10: N97.0–N97.9 |
| Ectopic pregnancy | ICD-8: 631.0–631.9 ICD-10: O00.0–O00.9 |
| Pregnancies with abortive outcome | ICD-8: 640.0–645.9 ICD-10: O01.0–O08.9 |
| Other complications of pregnancy or delivery | ICD-8: 630.0–639.9   ICD-8: 651.0–666.9  ICD-8: 670.0–678.9 ICD-10: O10.0–O16.9 ICD-10: O20.0–O48.9 ICD-10: O60.0–O75.9 ICD-10: O81.0–O99.9 |
| Delivery without mention of complication | ICD-8: 650.0–650.9 ICD-10: O80.0–O80.9 |
| Conditions originating in the perinatal period | ICD-8: 760.0–773.9 ICD-8: 776.0–779.9 ICD-10: P00.0–P54.9 ICD-10: P56.0–P96.9 |
| Hemolytic disease of fetus and newborn | ICD-8: 774.0–775.9 ICD-10: P55.0–P55.9 |
| Spina bifida and congenital hydrocephalus | ICD-8: 741.0–742.9 ICD-10: Q05.0–Q05.9 |
| Congenital malformations of the circulatory system | ICD-8: 746.0–747.9  ICD-10: Q20.0–Q28.9 |
| Cleft lip and cleft palate | ICD-8: 749.0–749.9 ICD-10: Q35.0–Q37.9 |
| Other congenital malformations of the digestive system | ICD-8: 750.0–750.0 ICD-8: 750.0–750.9  ICD-8: 751.0–751.9 ICD-10: Q38.0–Q40.9 ICD-10: Q42.0–Q45.9 |
| Absence, atresia and stenosis of small intestine | ICD-8: 750.0–750.1 ICD-10: Q41.0–Q41.9 |
| Other malformations of the genitourinary system | ICD-8: 752.2–753.9 ICD-10: Q50.0–Q52.9 ICD-10: Q54.0–Q64.9 |
| Undescended testicle | ICD-8: 752.0–752.1 ICD-10: Q53.0–Q53.9 |
| Congenital deformities of hip | ICD-8: 755.0–755.6 ICD-10: Q65.0–Q65.9 |
| Congenital deformities of feet | ICD-8: 754.0–754.9 ICD-10: Q66.0–Q66.9 |
| Other and unspecified congenital anomalies | ICD-8: 740.0–740.9 ICD-8: 743.0–745.9 ICD-8: 748.0–748.9 ICD-8: 757.0–759.9 ICD-10: Q00–Q04.9 ICD-10:Q06.0–Q07.9 ICD-10: Q10–Q18.9 ICD-10: Q30.0–Q34.9 ICD-10: Q80.0–Q99.9 |
| Other congenital malformations and deformations of the musculoskeletal system | ICD-8: 755.0–755.9 ICD-8: 755.0–756.9 ICD-8: 756.0–756.9  ICD-10: Q67.0–Q79.9 |
| Abdominal and pelvic pain | ICD-8: 785.0–785.5 ICD-10: R10.0–R10.9 |
| Senility | ICD-8: 794.0–794.9 ICD-10: R54.0–R54.9 |
| ^a^Based on WHO Tabulation List for Morbidity | |
