# Supplementary material for: Hospital Recorded Morbidity and Breast Cancer Incidence: A Nationwide Population-Based Case-Control Study
Source: PLoS One. 2012 Oct 19;7(10):e47329. doi: 10.1371/journal.pone.0047329 (PMC3477157; doi:10.1371/journal.pone.0047329)
Supplement: Table S3 — Morbidity categories preceding breast cancer diagnosis, number of exposed cases, corresponding odds ratios (ORs) and Empirical-Bayes-adjusted estimates accompanied by 95% CI for associations between morbidity categories and breast cancer incidence. (DOC) [file pone.0047329.s003.doc]

| **Supporting Information Table 3. Morbidity categories preceding breast cancer diagnosis, number of exposed cases, corresponding odds ratios (ORs) and Empirical-Bayes-adjusted estimates accompanied by 95%CI in parentheses for associations between morbidity categories and breast cancer incidence.** | | | |
| --- | --- | --- | --- |
| **Preceding comorbid disease category** | **# exposed cases** | **Original**  **estimates** | **Empirical-Bayes adjusted estimates** |
| Iron deficiency anemia | 32 | 0.61 (0.45, 0.81) | 0.91 (0.73, 1.15) |
| Chronic disease of tonsils and adenoids | 59 | 0.74 (0.52, 1.06) | 0.99 (0.78, 1.25) |
| Dementia | 5 | 0.77 (0.59, 1.00) | 0.96 (0.77, 1.20) |
| Malignant neoplasm of bone and articular cartilage | 50 | 0.77 (0.31, 1.91) | 1.05 (0.82, 1.33) |
| Other tuberculosis | 10 | 0.78 (0.41, 1.48) | 1.04 (0.81, 1.32) |
| Other anemias | 133 | 0.78 (0.66, 0.94) | 0.91 (0.74, 1.13) |
| Alzheimer’s disease | 18 | 0.79 (0.49, 1.28) | 1.02 (0.81, 1.30) |
| Other diseases of the bone | 104 | 0.83 (0.68, 1.01) | 0.95 (0.77, 1.19) |
| Other malignant neoplasms of urinary tract | 16 | 0.85 (0.51, 1.41) | 1.03 (0.81, 1.31) |
| Septicemia | 53 | 0.85 (0.64, 1.12) | 0.99 (0.79, 1.25) |
| Influenza | 39 | 0.85 (0.61, 1.18) | 1.01 (0.80, 1.27) |
| Hepatitis | 19 | 0.86 (0.54, 1.37) | 1.03 (0.81, 1.31) |
| Migraine | 114 | 0.86 (0.71, 1.04) | 0.96 (0.78, 1.19) |
| Osteoporosis with and without fracture | 369 | 0.87 (0.78, 0.96) | 0.92 (0.75, 1.11) |
| Rheumatoid arthritis and other inflammatory polyarthropathies | 438 | 0.88 (0.80, 0.98) | 0.92 (0.76, 1.12) |
| Gastric and duodenal ulcer | 467 | 0.89 (0.81, 0.98) | 0.93 (0.77, 1.12) |
| Acute myocaridal infarction | 429 | 0.89 (0.81, 0.99) | 0.93 (0.76, 1.13) |
| Benign neoplasm of brain and other parts of central nervous system | 45 | 0.90 (0.67, 1.23) | 1.02 (0.81, 1.28) |
| Epilepsy | 185 | 0.91 (0.78, 1.05) | 0.97 (0.79, 1.19) |
| Malignant neoplasm of larynx | 47 | 0.91 (0.49, 1.70) | 1.05 (0.82, 1.33) |
| Other acute upper respiratory infections | 59 | 0.91 (0.70, 1.19) | 1.01 (0.81, 1.27) |
| Gastritis and duodenitis | 353 | 0.92 (0.82, 1.02) | 0.95 (0.79, 1.16) |
| A-vitaminosis and other nutritional deficiency | 21 | 0.93 (0.59, 1.45) | 1.04 (0.82, 1.32) |
| Other infectious and parasitic diseases | 112 | 0.94 (0.77, 1.14) | 1.00 (0.81, 1.24) |
| Chronic sinusitis | 61 | 0.94 (0.72, 1.22) | 1.02 (0.81, 1.28) |
| Leukemia | 28 | 0.94 (0.64, 1.38) | 1.04 (0.82, 1.31) |
| Congenital deformations of hip | 12 | 0.94 (0.52, 1.71) | 1.05 (0.83, 1.33) |
| Cataract and other disorders of lens | 164 | 0.95 (0.89, 1.01) | 0.96 (0.80, 1.15) |
| Glaucoma | 1210 | 0.95 (0.81, 1.12) | 1.00 (0.81, 1.23) |
| Other peripheral vascular disease | 82 | 0.96 (0.76, 1.20) | 1.02 (0.82, 1.27) |
| Malignant neoplasm of stomach | 11 | 0.96 (0.52, 1.78) | 1.05 (0.83, 1.33) |
| Inflammatory diseases of *cervix uteri* | 14 | 0.96 (0.55, 1.66) | 1.05 (0.83, 1.33) |
| Other diseases of oesophagus, stomach and duodenum | 710 | 0.96 (0.89, 1.04) | 0.97 (0.81, 1.17) |
| Other congenital malformations and deformations of the musculoskeletal system | 45 | 0.96 (0.71, 1.31) | 1.03 (0.82, 1.30) |
| Other ischemic heart disease | 403 | 0.96 (0.87, 1.07) | 0.99 (0.81, 1.20) |
| Other endocrine, nutritional, and metabolic disorders | 492 | 0.97 (0.88, 1.06) | 0.99 (0.82, 1.19) |
| Female genital prolapse | 882 | 0.97 (0.90, 1.03) | 0.98 (0.81, 1.18) |
| Infections of the skin and subcutaneous tissue | 746 | 0.97 (0.88, 1.08) | 1.00 (0.82, 1.21) |
| Other disease of the nervous system | 861 | 0.98 (0.91, 1.05) | 0.99 (0.82, 1.19) |
| Benign neoplasm of kidney and other urinary organs | 81 | 0.98 (0.78, 1.23) | 1.03 (0.83, 1.28) |
| Other disorders of joints | 746 | 0.98 (0.91, 1.06) | 0.99 (0.83, 1.20) |
| Ectopic pregnancy | 24 | 0.99 (0.65, 1.50) | 1.05 (0.83, 1.32) |
| Congenital malformations of the circulatory system | 25 | 0.99 (0.66, 1.49) | 1.05 (0.83, 1.32) |
| Other and unspecified congenital anomalities | 78 | 0.99 (0.79, 1.25) | 1.03 (0.83, 1.29) |
| Atherosclerosis | 347 | 0.99 (0.89, 1.11) | 1.01 (0.83, 1.23) |
| Salpingitis and oophoritis | 75 | 0.99 (0.78, 1.26) | 1.03 (0.83, 1.29) |
| Other mental and behavioral disorders | 65 | 0.99 (0.77, 1.28) | 1.04 (0.83, 1.30) |
| Acquired deformities of limbs | 447 | 1.00 (0.90, 1.10) | 1.01 (0.83, 1.22) |
| Paralytic ileus and intestinal obstruction without hernia | 152 | 1.00 (0.85, 1.18) | 1.03 (0.83, 1.27) |
| Hernia | 647 | 1.00 (0.92, 1.08) | 1.01 (0.84, 1.22) |
| Hemorrhoids | 356 | 1.00 (0.90, 1.12) | 1.02 (0.84, 1.24) |
| Transient cerebral ischemic attacks and related syndromes | 301 | 1.01 (0.89, 1.13) | 1.02 (0.84, 1.25) |
| Pneumonia | 636 | 1.01 (0.93, 1.09) | 1.02 (0.84, 1.23) |
| Crohn’s disease and ulcerative colitis | 183 | 1.01 (0.87, 1.18) | 1.03 (0.84, 1.27) |
| Malignant neoplasm of *cervix uteri* | 70 | 1.01 (0.79, 1.30) | 1.04 (0.84, 1.30) |
| Malignant neoplasm of other digestive organs and peritoneum | 13 | 1.02 (0.57, 1.80) | 1.05 (0.83, 1.34) |
| Pregnancies with abortive outcome | 383 | 1.02 (0.91, 1.13) | 1.03 (0.85, 1.25) |
| Urolithiasis/calculus of urinary system | 228 | 1.02 (0.89, 1.17) | 1.03 (0.84, 1.26) |
| Bronchitis, emphysema and other chronic obstructive pulmonary diseases | 937 | 1.02 (0.95, 1.09) | 1.02 (0.85, 1.23) |
| Other inflammatory diseases of eye | 334 | 1.02 (0.91, 1.14) | 1.03 (0.85, 1.26) |
| Other heart disease | 220 | 1.02 (0.89, 1.17) | 1.04 (0.85, 1.27) |
| Other diseases of arteries, arterioles, and capillaries | 195 | 1.02 (0.88, 1.18) | 1.04 (0.84, 1.27) |
| Varicose veins of lower extremities | 888 | 1.03 (0.96, 1.10) | 1.03 (0.86, 1.24) |
| Soft tissue disorders | 1765 | 1.03 (0.98, 1.08) | 1.03 (0.86, 1.23) |
| Other diseases of the teeth, oral cavity, salivary glands, and jaws | 368 | 1.03 (0.92, 1.14) | 1.04 (0.85, 1.26) |
| Hemorrhagic conditions and other diseases of blood and blood-forming organs | 76 | 1.03 (0.81, 1.30) | 1.05 (0.84, 1.31) |
| Essential (primary) hypertension | 734 | 1.03 (0.95,1.11) | 1.03 (0.86, 1.24) |
| Angina pectoris | 892 | 1.03 (0.96, 1.10) | 1.03 (0.86, 1.24) |
| Retinal detachments and breaks | 119 | 1.03 (0.85, 1.24) | 1.05 (0.84, 1.29) |
| Other diseases of the ear and mastoid process | 1942 | 1.03 (0.99, 1.09) | 1.04 (0.87, 1.24) |
| Other diseases of the musculoskeletal system and connective tissue | 472 | 1.04 (0.94, 1.14) | 1.04 (0.86, 1.26) |
| Cerebral infarction | 267 | 1.04 (0.91, 1.18) | 1.04 (0.85, 1.27) |
| Multiple sclerosis and other demyelinating diseases | 102 | 1.04 (0.85, 1.28) | 1.05 (0.85, 1.31) |
| Abdominal and pelvic pain | 1031 | 1.04 (0.98, 1.11) | 1.04 (0.87, 1.25) |
| Other diseases of the respiratory system | 221 | 1.05 (0.91, 1.20) | 1.05 (0.86, 1.29) |
| Acute bronchitis and acute bronchiolitis | 120 | 1.05 (0.87, 1.26) | 1.05 (0.85, 1.30) |
| Acute pancreatitis and other diseases of the pancreas | 146 | 1.05 (0.88, 1.24) | 1.05 (0.85, 1.30) |
| Acute pharyngitis and acute tonsilitis | 44 | 1.05 (0.77, 1.43) | 1.06 (0.84, 1.33) |
| Parkinson’s disease | 53 | 1.05 (0.79, 1.39) | 1.06 (0.84, 1.32) |
| Other diseases of upper respiratory tract | 365 | 1.05 (0.94, 1.17) | 1.05 (0.87, 1.28) |
| Other diseases of the urinary system | 549 | 1.05 (0.96, 1.15) | 1.05 (0.87, 1.27) |
| Osteoarthritis and allied conditions | 2217 | 1.05 (1.00, 1.10) | 1.05 (0.88, 1.26) |
| Other diseases of the eye and adnexa | 766 | 1.05 (0.98, 1.13) | 1.05 (0.87, 1.27) |
| Diabetes mellitus | 663 | 1.05 (0.97, 1.14) | 1.05 (0.87, 1.27) |
| Cystitis | 359 | 1.05 (0.95, 1.18) | 1.06 (0.87, 1.28) |
| Other diseases of the skin and subcutaneous tissue | 828 | 1.06 (0.98, 1.13) | 1.06 (0.88, 1.27) |
| Other cerebrovascular disease | 592 | 1.06 (0.97, 1.16) | 1.06 (0.88, 1.28) |
| Cervical and other intervertebral disc disorders | 1550 | 1.06 (1.01, 1.12) | 1.06 (0.89, 1.27) |
| Other inflammatory diseases of female pelvic organs | 204 | 1.06 (0.92, 1.23) | 1.06 (0.87, 1.30) |
| Cholelithiasis and cholecystitis | 892 | 1.07 (1.00, 1.14) | 1.07 (0.89, 1.28) |
| Malignant neoplasm of rectosigmoideum junction, rectum, anus, and anal canal | 84 | 1.07 (0.85, 1.34) | 1.06 (0.85, 1.32) |
| Other disorders of genitourinary tract | 1155 | 1.08 (1.01, 1.14) | 1.07 (0.90, 1.29) |
| Malignant neoplasm of bladder | 41 | 1.08 (0.78, 1.49) | 1.06 (0.84, 1.34) |
| Other bacterial disease | 169 | 1.08 (0.92, 1.27) | 1.07 (0.87, 1.32) |
| Infections of kidney | 157 | 1.08 (0.92, 1.28) | 1.07 (0.87, 1.32) |
| Other diseases of the digestive system | 1520 | 1.08 (1.03, 1.14) | 1.08 (0.90, 1.29) |
| Other viral diseases | 167 | 1.09 (0.93, 1.28) | 1.07 (0.87, 1.32) |
| Other disorders of ovary, fallopian tube and parametrium | 259 | 1.09 (0.96, 1.24) | 1.08 (0.88, 1.34) |
| Carcinoma *in situ* of *cervix uteri* | 199 | 1.09 (0.94, 1.26) | 1.08 (0.88, 1.32) |
| Other dorsopathies | 352 | 1.09 (0.98, 1.22) | 1.08 (0.89, 1.32) |
| Other malignant neoplasms of lymphoid, hematopoietic, and related tissue | 81 | 1.10 (0.87, 1.38) | 1.07 (0.86, 1.33) |
| Benign neoplasm of the skin | 167 | 1.11 (0.94, 1.30) | 1.08 (0.88, 1.33) |
| Malignant neoplasm of trachea, bronchus, and lung | 47 | 1.11 (0.82, 1.50) | 1.07 (0.85, 1.34) |
| Endometriosis | 121 | 1.11 (0.92, 1.34) | 1.08 (0.87, 1.34) |
| Alcohol-, drug-abuse-related disease | 280 | 1.11 (0.98, 1.26) | 1.10 (0.90, 1.34) |
| Congenital deformations of feet | 23 | 1.12 (0.73, 1.72) | 1.07 (0.84, 1.35) |
| Diseases of appendix | 252 | 1.12 (0.98, 1.28) | 1.10 (0.90, 1.34) |
| Iodine-deficiency-related thyroid disorders | 490 | 1.12 (1.02, 1.23) | 1.11 (0.92, 1.34) |
| Neurotic, stress-related, and somatoform disorders | 130 | 1.13 (0.94, 1.35) | 1.09 (0.88, 1.35) |
| Malignant neoplasm of other genitourinary organs | 13 | 1.13 (0.64, 2.01) | 1.06 (0.84, 1.35) |
| Disorders of menstruation | 2007 | 1.13 (1.08, 1.19) | 1.13 (0.94, 1.35) |
| Acute rheumatic fever | 5 | 1.14 (0.45, 2.87) | 1.06 (0.83, 1.35) |
| Respiratory tuberculosis | 15 | 1.15 (0.67, 1.95) | 1.07 (0.84, 1.35) |
| Phlebitis, thrombophlebitis, venous embolism and thrombosis | 184 | 1.15 (1.01, 1.30) | 1.12 (0.92, 1.36) |
| Other diseases of the circulatory system | 106 | 1.15 (0.94, 1.41) | 1.10 (0.88, 1.36) |
| Other disorders of thyroid | 646 | 1.16 (1.06, 1.25) | 1.14 (0.94, 1.37) |
| Osteochrondrosis | 35 | 1.16 (0.81, 1.64) | 1.08 (0.85, 1.36) |
| Depression | 88 | 1.16 (0.93, 1.44) | 1.09 (0.88, 1.36) |
| Chronic rheumatic heart disease | 44 | 1.16 (0.85, 1.59) | 1.08 (0.86, 1.36) |
| Other diseases of liver and gallbladder | 193 | 1.17 (1.01, 1.36) | 1.12 (0.91, 1.38) |
| Rheumatism | 342 | 1.17 (1.05, 1.31) | 1.14 (0.93, 1.38) |
| Inflammatory disease of the central nervous system | 27 | 1.17 (0.79, 1.75) | 1.08 (0.85, 1.36) |
| Strabismus | 81 | 1.18 (0.94, 1.49) | 1.10 (0.88, 1.37) |
| Other complications of pregnancy or delivery | 280 | 1.18 (1.04, 1.34) | 1.14 (0.93, 1.39) |
| Malignant neoplasm of skin | 290 | 1.18 (1.05, 1.34) | 1.14 (0.93, 1.39) |
| Malignant neoplasm of other specified sites | 34 | 1.19 (0.83, 1.70) | 1.08 (0.86, 1.36) |
| Benign neoplasm of ovary | 345 | 1.20 (1.07, 1.34) | 1.15 (0.95, 1.40) |
| Leiomyoma of uterus | 931 | 1.21 (1.13, 1.29) | 1.18 (0.99, 1.42) |
| Diarrhea and gastro-enteritis of presumed infectious origin | 269 | 1.21 (1.06, 1.37) | 1.15 (0.94, 1.41) |
| Other malignant neoplasms of respiratory and intrathoracic organs | 8 | 1.21 (0.58, 2.52) | 1.07 (0.84, 1.36) |
| Pulmonary embolism | 94 | 1.22 (0.98, 1.51) | 1.12 (0.90, 1.39) |
| Malignant neoplasm of other unspecified parts of uterus | 190 | 1.22 (1.05, 1.42) | 1.15 (0.93, 1.41) |
| Bronchiestasis | 21 | 1.22 (0.78, 1.92) | 1.08 (0.85, 1.36) |
| Other malignant neoplasm of female genital organs | 121 | 1.22 (1.01, 1.48) | 1.13 (0.91, 1.40) |
| Conditions originating in the perinatal period | 6 | 1.22 (0.52, 2.86) | 1.06 (0.84, 1.36) |
| Conduction disorders and cardiac arrhythmias | 998 | 1.23 (1.15, 1.31) | 1.20 (1.00, 1.44) |
| Intracranial hemorrhage | 114 | 1.23 (1.01, 1.50) | 1.13 (0.91, 1.40) |
| Congestive heart failure | 469 | 1.24 (1.12, 1.36) | 1.19 (0.98, 1.44) |
| Delivery without mention of complication | 217 | 1.24 (1.07, 1.43) | 1.16 (0.95, 1.42) |
| Other intestinal infectious disease | 79 | 1.26 (1.00, 1.60) | 1.13 (0.90, 1.41) |
| Malignant neoplasm of colon | 171 | 1.27 (1.08, 1.48) | 1.17 (0.95, 1.44) |
| Mood affective disorders | 36 | 1.31 (0.93, 1.85) | 1.10 (0.88, 1.39 |
| Other *in situ* and benign neoplasms and neoplasms of uncertain and unknown behaviour | 2179 | 1.32 (1.26, 1.38) | 1.30 (1.09, 1.55) |
| Female infertility | 48 | 1.32 (0.98, 1.79) | 1.12 (0.89, 1.40) |
| Malignant neoplasm of other, ill-defined, secondary, unspecified and multiple sites | 69 | 1.33 (1.03, 1.70) | 1.14 (0.91, 1.42) |
| Osteomyelitis and periostitis | 27 | 1.37 (0.92, 2.05) | 1.10 (0.87, 1.39) |
| Nephritis and nephrosis | 44 | 1.38 (1.01, 1.89) | 1.13 (0.89, 1.42) |
| Myostitis | 15 | 1.39 (0.81, 2.38) | 1.09 (0.86, 1.38) |
| Hodgkin’s disease | 11 | 1.39 (0.74, 2.62) | 1.08 (0.85, 1.37) |
| Schizophrenia, schizotypal, and delusional disorders | 31 | 1.51 (1.04, 2.21) | 1.13 (0.89, 1.42) |
| Malignant neoplasm of lip, oral cavity and pharynx | 46 | 1.51 (1.11, 2.07) | 1.15 (0.92, 1.45) |
| Other congenital malformations of the digestive system | 26 | 1.56 (1.03, 2.35) | 1.12 (0.89, 1.42) |
| Other malformations of the genitourinary system | 49 | 1.62 (1.20, 2.19) | 1.18 (0.94, 1.48) |
| Disorders of breast^a^ | 1052 | 1.62 (1.52, 1.73) | 1.54 (1.28, 1.84) |
| Acute poliomyelitis | 18 | 1.94 (1.17, 3.21) | 1.13 (0.89, 1.43) |
| *Pooled effect estimate* |  | *1.07 (1.06, 1.08)* | *1.06 (1.04, 1.08)* |
| **Abbreviations.** OR: odds ratio, # exp cases: number of exposed cases **^a^**Disorders of the breast consist of benign mammary dysplasia, inflammatory disorders of breast, hypertrophy of breast, unspecified lump in breast, and other disorders of breast | | | |
